# Limbal Epithelial Stem Cells in the Diabetic Cornea

**DOI:** 10.3390/cells12202458

**Published:** 2023-10-16

**Authors:** Lamia Nureen, Nick Di Girolamo

**Affiliations:** School of Biomedical Sciences, Faculty of Medicine and Health, University of New South Wales, Sydney, NSW 2052, Australia; l.nureen@unsw.edu.au

**Keywords:** limbal epithelial stem cells, diabetic keratopathy, diabetic corneal disease, limbus

## Abstract

Continuous replenishment of the corneal epithelium is pivotal for maintaining optical transparency and achieving optimal visual perception. This dynamic process is driven by limbal epithelial stem cells (LESCs) located at the junction between the cornea and conjunctiva, which is otherwise known as the limbus. In patients afflicted with diabetes, hyperglycemia-induced impairments in corneal epithelial regeneration results in persistent epithelial and other defects on the ocular surface, termed diabetic keratopathy (DK), which progressively diminish vision and quality of life. Reports of delayed corneal wound healing and the reduced expression of putative stem cell markers in diabetic relative to healthy eyes suggest that the pathogenesis of DK may be associated with the abnormal activity of LESCs. However, the precise role of these cells in diabetic corneal disease is poorly understood and yet to be comprehensively explored. Herein, we review existing literature highlighting aberrant LESC activity in diabetes, focusing on factors that influence their form and function, and emerging therapies to correct these defects. The consequences of malfunctioning or depleted LESC stocks in DK and limbal stem cell deficiency (LSCD) are also discussed. These insights could be exploited to identify novel targets for improving the management of ocular surface complications that manifest in patients with diabetes.

## 1. Introduction

Diabetes mellitus (commonly known as diabetes) is a metabolic disorder characterized by prolonged elevation of blood glucose or hyperglycemia. Its two main forms include type 1 diabetes (T1D) where chronic hyperglycemia results from the autoimmune destruction of pancreatic β-cells, and type 2 diabetes (T2D) which features defective insulin secretion or sensitivity [1]. If left uncontrolled, both forms can lead to debilitating comorbidities, including cardiovascular disease, chronic kidney failure, peripheral nerve damage and loss of vision [2], thereby diminishing quality of life. The prevalence of diabetes has become a major public health concern as it approaches epidemic proportions at an alarming rate. An estimated 537 million adults worldwide are currently diagnosed with this disease with numbers projected to exceed 700 million within the next 20 years [3]. 

Diabetes increases the likelihood of visual impairment by approximately 3-fold and is the leading cause of preventable blindness in working-age adults [4]. Ocular complications such as diabetic retinopathy (DR), clinically characterized by macular edema, microaneurysms, hemorrhages, and neovascularization of the retina, affect over 33% of all patients with diabetes [5,6]. Therefore, due to its irreversible impacts on vision [7], DR remains the primary target for the treatment of diabetic eye disease. However, the prevalence of severe diabetes-related abnormalities manifesting on the cornea is 2-fold higher compared to DR, affecting up to 70% of patients with diabetes worldwide [8,9]. Diabetic corneal disruptions can be under-diagnosed and present as mild changes, including persistent epithelial defects (diabetic keratopathy; DK), damage to nerve fibers (diabetic corneal neuropathy; DCN), endothelial dysfunction, altered stromal composition, alterations in the tear film as well as biomechanical abnormalities imposed by oxidative stress and exposure to hyperglycemia [10,11,12,13,14,15]. More severe clinical manifestations include the development of non-healing neurotrophic ulcers, perforations, loss of corneal sensitivity, recurrent erosions, significant delays in re-epithelialization and ocular surface oedema over time, ultimately resulting in vision loss [14,15,16]. Importantly, DK is the most frequent underlying condition, affecting up to 64% of patients with diabetic corneal disease [9]. 

The current management of DK is complicated by the paucity of diagnostic markers and is largely reliant on symptom-specific treatments such as topical ointments and antibiotics, owing to its complex pathophysiology and the limited number of clinical studies [14,17]. As chronic hyperglycemia delays corneal wound healing [18,19,20,21], patients with diabetes are at a heightened risk of developing post-operative complications from standard surgical interventions, including vitrectomy for retinopathy [22], corneal transplantation for keratopathy [23], and cataract removal or refractive surgery for vision correction [24,25]. Additionally, treatment options for diabetics are limited due to their increased susceptibility to other sight-threatening complications, including ocular ischemic syndrome, optic neuropathy and glaucoma [26]. Given the rapid increase in diabetes prevalence, concomitant with the escalating incidence of diabetes-associated ocular surface diseases [27], mechanistic investigations pertaining to corneal pathophysiology during metabolic imbalance are warranted to assist the development and implementation of novel diagnostics, therapeutics and measures of treatment outcome.

Limbal epithelial stem cells (LESCs) are the quintessential hierarchical cellular source for the lifelong renewal and replenishment of the corneal epithelium [28]. When the cornea is challenged within its confines, LESC proliferation is ramped in its outer limits, causing population pressure to propel new progeny into the defective site [29]. In this context, the wound is sealed in a timely manner, thus preventing microbial colonization and other complications, after which maturation and stratification of the epithelium completes the regenerative process [28,30]. Aberrations to LESCs can impose adverse effects on corneal function, resulting in opacity and vision loss. Notably, experimental evidence of diminished LESCs, defective expression of putative LESC markers and delayed corneal epithelial wound healing in diabetic compared to otherwise healthy eyes [19,21,31,32,33,34] suggests that LESCs are involved in the pathogenesis of DK. Thus far, reduced corneal sensitivity and immune dysregulation have been identified as major causes of delayed epithelial repair in corneas of diabetic individuals [16,18,35,36,37]. Stem cell (SC) therapy, including mesenchymal and hematopoietic SC administration, can also limit the progression of corneal epithelial abnormalities in animal models of diabetes [38,39]. However, the effect of metabolic imbalance on LESCs and the mechanisms by which this contributes to corneal epithelial defects in DK remain an enigma.

Herein, evidence demonstrating the loss or dysfunction of LESCs in the diabetic cornea is evaluated to determine the mechanisms by which they impinge on corneal health, become defective under hyperglycemic conditions, and therefore contribute to the pathogenesis of DK. Because LESCs play a beneficial role in corneal wound healing and epithelial regeneration, bridging the current knowledge gap concerning factors that trigger pathological processes leading to diabetic corneal disease will provide novel insights toward developing innovative clinical tools for early diagnosis and the effective management of ocular surface complications in patients with diabetes. 

## 2. Overview of LESC Functional Dynamics

The cornea is a transparent tissue which comprises the outermost layer of the eye and consists of non-keratinized stratified squamous epithelium on its anterior surface. It forms a protective barrier against noxious external agents and acts as a clear avascular window for projecting light onto the retina, thereby facilitating optimal visual perception. These structural and functional attributes are reliant on LESCs, which constitute a rare population of total ocular surface epithelia compartmentalized in the basal layer of a highly vascularized and innervated zone between the cornea and conjunctiva otherwise known as the limbus [40,41,42]. LESCs have attracted ample attention due to their intrinsic replicative and regenerative capabilities, enabling them to repair damaged corneal epithelia and replenish aged cells that are exfoliated from the corneal surface throughout life [28]. 

Under steady state, cellular attrition in the superficial layer of the central corneal epithelium prompts LESCs to replace lost cells by spawning progeny that can enter a differentiation program. Typically, asymmetric division prevails whereby a LESC divides to generate two daughter cells: a newly formed LESC and an early transient amplifying cell (TAC) [43]. Symmetric division also occurs but is less frequent. LESCs exhibit lifelong self-renewal capacity and are therefore spared from undergoing differentiation. Early TACs can be difficult to distinguish (morphologically and phenotypically) from their ancestral SCs. However, once evicted from their basal location, they change in phenotype and migrate centripetally toward the central cornea as mature TACs [29]. Cells that depart from the niche are short lived and gradually lose their proliferative potential, eventually becoming terminally differentiated cells that are shed from the corneal surface [44,45]. In response to injury, LESCs receive signals that trigger their proliferation and allow their progeny to migrate to the affected site, thereby ensuring that wounds are resurfaced in a timely manner. Recently, two distinct populations of active and quiescent SCs were identified within the inner and outer limbal sanctum [46,47], each with the capacity to differentially regulate corneal epithelial homeostasis and wound healing, depending on the scenario [48,49]. 

LESC activity is also influenced by their immediate microenvironment. This comprises (but is not limited to) the underlying basement membrane, stroma and its supporting cells, and the neurovascular network, which are collectively referred to as the LESC niche [50]. In humans, LESCs are housed within the basal layer of a series of fibrovascular ridges that decorate the limbus, which are termed the Palisades of Vogt (POV) [51]. This feature ensures they are shielded from external environmental insults whilst remaining proximal to the niche to effectively receive nutrients and chemical inputs from their surroundings [52,53]. LESCs are also nestled in other structures known as the limbal epithelial crypts and pits, which resemble a solid cord of cells extending tangentially or circumferentially from the peripheral aspect of the POV [54,55]. In contrast, the mouse limbus is relatively unremarkable with no such distinguishing features [28]. In addition to the anatomical location and topographical features, biochemical mediators, including various growth factors, differentiation factors, peptides, cytokines, chemokines, and integrins as well as neurotrophic and metabolic factors that percolate from niche components including the extracellular matrix (ECM), limbal blood vessels, mesenchymal cells, adjacent conjunctival epithelia, and ocular surface neurons, also play a vital role in LESC maintenance [50,56,57]. External influences may include environmental stressors such as chemical pollutants, thermal burns, ultraviolet radiation, toxic gases, and microbes, all of which trigger inflammation and neovascular responses within the cornea and therefore alter regulatory mechanisms initiated by the niche [58,59,60]. 

An inadequate number or depleted LESC stocks can result in the development of a vision-threatening disease known as limbal stem cell deficiency (LSCD). Under these conditions, the barrier function imposed by the limbus is breached and the corneal epithelium is unable to self-renew, thereby triggering a pathological wound-healing response which involves the invasion of an inflamed fibrovascular pannus of conjunctival cells into the cornea (otherwise known as conjunctivalization), resulting in a compromised ocular surface [61]. Significant, albeit less severe pathological consequences, such as decreased LESC activity, disintegration of the POV and delayed epithelial regeneration can arise in diabetes [21,32,34,62]. 

## 3. LESC Modifications in the Diabetic Cornea

**Figure 1:**
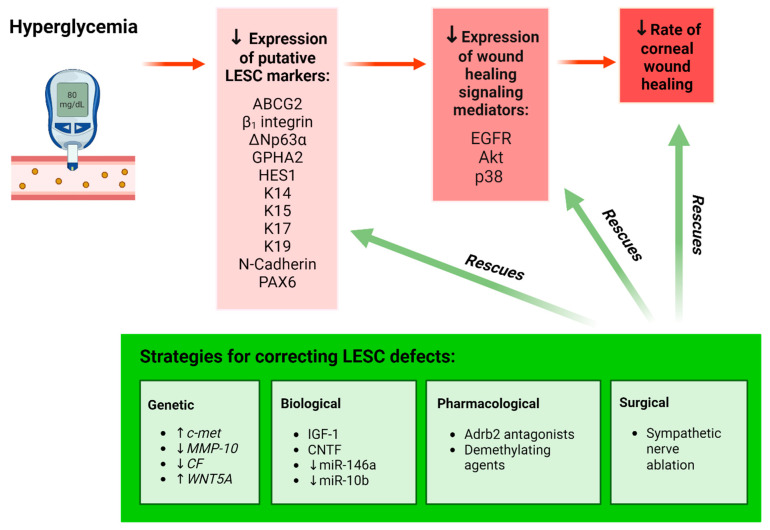
Schematic representation of the effect of hyperglycemia on LESCs. Hyperglycemia results in a significant reduction in the expression of putative LESC markers and the number of cells positive for these markers in the limbus, resulting in delayed corneal wound healing (red highlight). Various genetic, biological, pharmacological and surgical interventions have been employed to rescue LESC function and enhance wound healing in organ-cultured diabetic human corneas, cultured diabetic LECs and mouse models of diabetes (green highlight). Upward arrow (↑) represents “increasing the amount of”; Downward arrow (↓) represents “decreasing the amount of”. Figure generated with BioRender.com.

Because of their pivotal role in supporting the corneal epithelium, especially during wound resolution, it is postulated that functional abnormalities in LESCs account for persistent epithelial defects that manifest on the diabetic ocular surface. To date, there have been few independent reports on the effect of diabetes on LESC dynamics [32,33,34,62]. The same studies exploit changes in putative LESC marker expression as surrogate measures of their density and function in diabetes. Moreover, investigations that ascertain the effects of gene therapy on the regulation of corneal wound healing [63,64,65] provide additional insights into the mechanisms that influence LESC phenotype under hyperglycemic conditions (Figure 1). 

**Figure 2:**
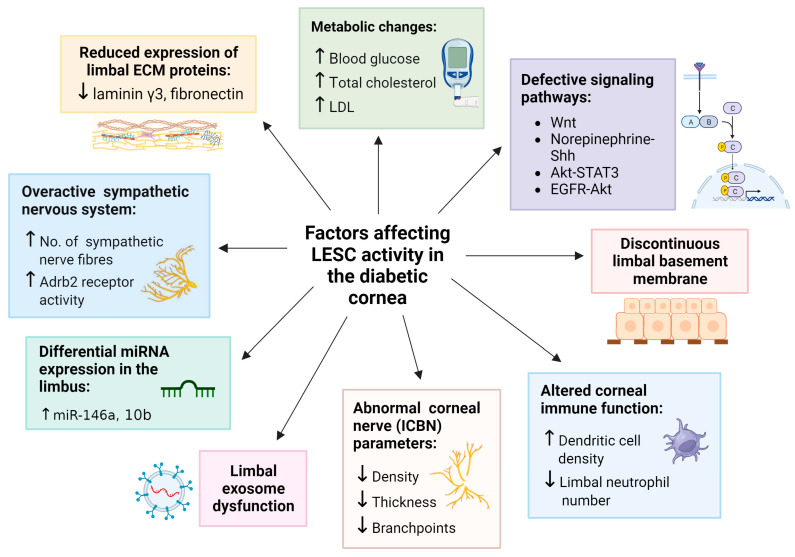
Factors which influence LESC function in diabetes. Each factor is associated with a significant reduction in the expression of putative LESC markers in studies that employ organ-cultured diabetic human corneas, diabetic LECs and mouse models of diabetes. Upward arrow (↑) represents “increase in”; Downward arrow (↓) represents “decrease in”. Figure generated with BioRender.com.

A direct association between diabetes and LESC marker expression was first demonstrated in tissue sections from organ-cultured human corneas [34]. Compared to healthy counterparts, corneas from patients with diabetes exhibited significantly reduced immunoreactivity and number of cells positive for proteins that are commonly used to identify LESCs, including ATP-binding cassette superfamily G member 2 (ABCG2), neural cadherin (N-Cadherin), ∆Np63α, keratin 15 (K15), K17, K19 and β_1_ integrin, thereby hinting that these cells are either depleted or dysfunctional under disease-bearing conditions [34]. Notably, such alterations coincided with structural abnormalities, including a discontinuous epithelial basement membrane and reduced expression of the ECM proteins, laminin γ3 and fibronectin, in the diabetic limbus [34] (Figure 2). Suppressed expression of numerous putative LESC markers has also been detected in cultured LESC-containing limbal epithelial cells (LECs) isolated from diabetic compared to normal cadaveric human corneoscleral rims [63]. Most recently, clinical examination of the limbal architecture using in vivo confocal microscopy (IVCM) revealed a significant loss or absence of the LESC-harboring POV in patients with T2D compared to healthy individuals [62], providing further circumstantial evidence that LESCs may be depleted in this disease. Alterations in LESC density was further correlated with duration of hyperglycemia, changes in total cholesterol and low-density lipoprotein (LDL) levels [62] (Figure 2). Therefore, modifications in limbal anatomy and components of its microenvironment due to metabolic imbalances negatively impact LESC density and activity in the diabetic cornea.

**Figure 3:**
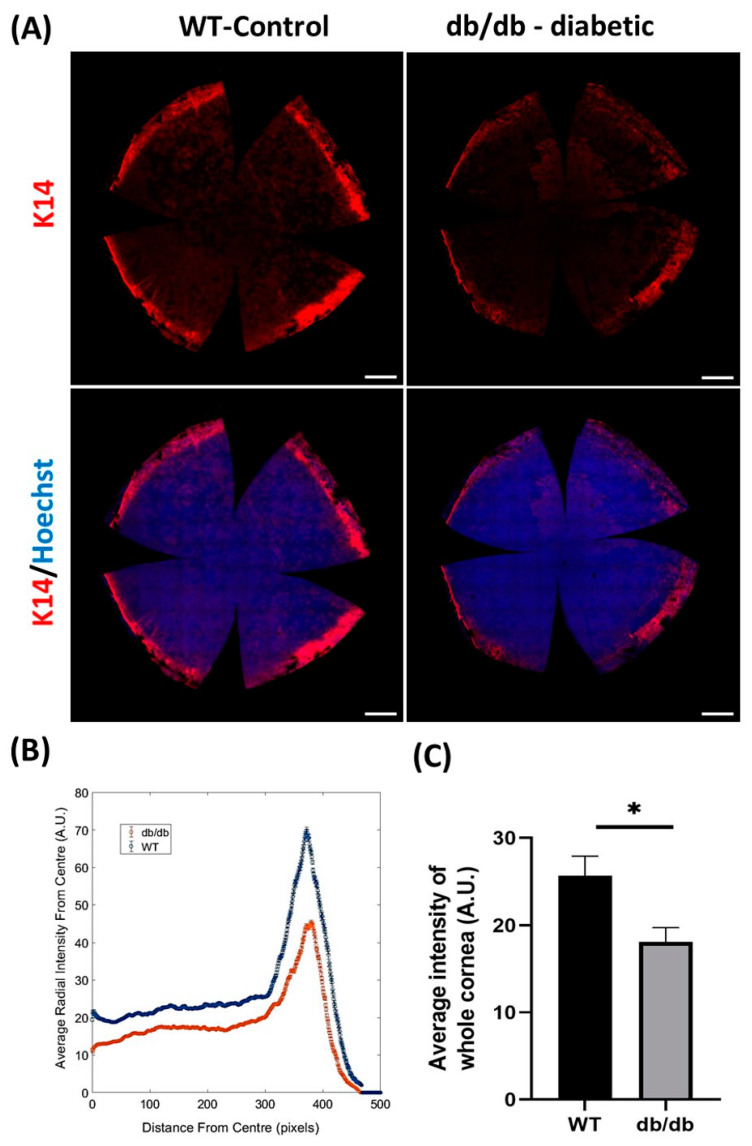
Measurement of K14 immunofluorescence staining intensity in db/db and WT control murine corneas. Intensity for K14 was measured in whole corneas obtained from 32-week-old db/db (*n = 6*) and WT (*n = 6*) mice after imaging with a scanning confocal microscope with 20× objective lens. (**A**) Representative images of K14 immunofluorescence staining (red) in whole-mount WT and db/db mouse corneas. Hoechst (blue) was used to visualize cell nuclei. (**B**) Comparison of average K14 staining intensity profile relative to distance (in pixels) from center of each whole cornea. Individual values represent average radial intensity at a specific distance (in pixels) from the center. Peak profile on graph represents intensity of K14 signal in the limbal region of db/db (red) and WT (blue) corneas. (**C**) Computed average K14 intensity in corneas is significantly decreased in db/db compared to WT corneas. All images represent the maximum intensity projection of epithelial layers. Statistical significance was determined using an unpaired Student’s *t*-test (* *p* < 0.05). Error bars represent ± standard error of mean. Scale bars represent 500 µm. All immunofluorescence procedures, microscopy and image acquisition were standardized and performed according to our previous reports [30,66].

Changes in LESC marker expression have also been observed in animal models of diabetes. Compared to age-matched wild-type (WT) controls, histological sections of corneas from mice with T2D (induced by homozygous leptin receptor-deficiency and identified by db/db genotype,) exhibited markedly reduced immunoreactivity and number of cells positive for HES1 and K19 proteins in basal limbal epithelia [33]. To overcome the limitations of surveying tissue sections (which only provide data from a confined region within a given tissue) and the subjectivity associated with manually grading immunostaining intensity (which many prior studies employ), we objectively quantified immunofluorescence intensity of the putative LESC marker, K14, in whole corneas of db/db mice using our previously published algorithm [30,66]. Using this modality, we computed a significant reduction in K14 protein expression in diabetic relative to WT corneas, which was most pronounced in the limbal zone (Figure 3). These findings corroborate previous immunohistochemical observations [33,34] as well as molecular data which demonstrate reduced mRNA expression for several LESC marker-associated genes including, *K15*, *Hes1* and *p75*, using real-time quantitative polymerase chain reaction (qPCR) in db/db mice [33]. Most recently, a reduction in both mRNA and protein expression for putative LESC markers, K15, ∆Np63α and glycoprotein hormone alpha-2 (GPHA2) (Figure 1) was detected in the basal limbal epithelia of corneas obtained from streptozotocin (STZ)-induced T1D mice [67]. Hence, the metabolic phenotypes of both T1D and T2D can alter LESC marker expression and content, indicating that their function is also affected. 

Evidence for LESC loss or dysfunction in diabetes has been strengthened through observations that elucidated the impact of this supposition on corneal wound healing (Figure 1). To this end, Kramerov and colleagues demonstrated that reduced expression of the LESC markers, paired box protein-6 (PAX6), ∆Np63α, K15, K17 and ABCG2 is concomitant with a 20% delay in scratch wound closure by primary LECs isolated from diabetic compared to normal human cadaveric corneoscleral rims [32]. This aberrant healing response is likely attributed to a hyperglycemia-induced modulation in cell movement, since supplementation with epidermal growth factor (EGF), a potent stimulator of cell migration, was unable to restore wound closure [32]. Therefore, delayed corneal wound healing in patients with diabetes resulting from impaired cell migration into the defective site is likely to be a consequence of debilitated LESCs.

Gene therapies have been instrumental in correcting LESC defects in the diabetic cornea (Figure 1, green box). Prior knowledge of differential gene expression, including significant downregulation of the hepatocyte growth factor receptor gene, *c-met,* and upregulation of proteinase-encoding genes such as matrix metalloproteinase-10 (*MMP-10*) and cathepsin F (*CF*) in human diabetic corneas [21,68], inspired researchers to develop manipulative approaches that specifically target these genes in the diabetic cornea. Accordingly, *c-met* overexpression promoted epithelial repair, re-established a basement membrane and restored LESC marker expression in ex vivo organ-cultured diabetic human corneas [34]. Similarly, small hairpin RNA (shRNA)-mediated, as well as non-toxic nanobiopolymer-based *c-met* overexpression and silencing of *MMP-10* and *CF* genes significantly accelerated wound closure, which is concurrent with increased ABCG2, ∆Np63α, K14, K15, K17 and laminin γ3 expression (Figure 1, green box) in organ-cultured diabetic corneas and diabetic LECs when compared to vector-transduced controls [64,69]. Moreover, gene therapy specifically directed to the limbal compartment was sufficient to improve wound healing and LESC marker expression at levels similar to transducing organ-cultured whole corneas ex vivo [65]. These observations highlight the functional significance of the limbal niche compartment in repairing corneal defects under diabetic conditions. Importantly, these investigations have also unearthed the involvement of wound-healing mediators, including phosphorylated forms of the EGF receptor (EGFR), Akt and p38, as well as associated cell signaling pathways in diabetic corneas (Figure 1). Expression of these mediators, including the rate of corneal wound healing, in conjunction with LESC marker expression, was markedly elevated following the simultaneous modification of these genes [65]. Collectively, these observations suggest that dysregulated signal-transduction intermediates are responsible for defective LESC activity during metabolic imbalance and that specifically targeting the limbal compartment can rescue LESC marker expression and wound resolution in diabetes.

In addition to conventional methodologies that include histology and two-dimensional cell culture, advanced molecular techniques have recently been employed to determine the LESC phenotype in diabetes. While most studies report modifications in LESC biomarker expression, transcriptional analyses using single-cell RNA sequencing (scRNA-seq) revealed unimpressive differences between diabetic and healthy murine corneas [70]. Specifically, no changes in mRNA transcripts for the LESC-associated genes *Krt14* and *Gpha2* were observed in STZ-induced T1D compared to the normal mouse corneal epithelium [70]. In this paradigm, cell heterogeneity was well preserved with only two genes (including one for LESCs) differentially expressed in diabetic verses healthy cells [70]. These results imply that a diabetes-associated loss of LESCs recorded by others [33,34,62] is not a consequence of a defective differentiation program. Moreover, given that these data were collated from uninjured eyes [70], it is possible that LESC dysfunction under hyperglycemia does not manifest until the cornea is perturbed. It is also conceivable that defective mRNA translation accounts for diminished LESC marker expression in diabetes. Notably, disparities between studies may be related to differences in species, animal models, the duration and severity of hyperglycemia, and the panel of gene and phenotypic markers as well as the biochemical and molecular investigations employed. 

Alternative explanations exist, which pertain to epigenetic changes pertinent to differences between humans and animal models of diabetes. Genome-wide DNA methylation of LESC-containing primary LECs exhibited significant hypermethylation of the *WNT5A* gene in cells isolated from diabetic compared to human cadaveric corneolimbal rims, suggesting its inactivation or downregulation [71]. This was confirmed when a pronounced reduction in WNT5A protein levels was recorded in the diabetic human limbal epithelium [71]. Importantly, WNT5A is localized to basal human limbal epithelia [72], suggesting that defective Wnt signaling may influence LESC function in diabetes (Figure 2). Accordingly, the modulation of Wnt signaling via demethylating agents, gene transduction of WNT5A regulators and exogenously dispensed WNT5A elicited significant improvements in limbal K15 and K17 expression as well as wound resolution (Figure 1) through promoting the phosphorylation of phospholipase C and protein kinase C and subsequent downstream activation of the non-canonical calcium signaling pathway in diabetic LECs and organ-cultured human corneas [71]. Interestingly, aberrant activity of Wnt ligands can also result from the diabetes-associated dysregulation of its receptors, since preliminary scRNA-seq followed by qPCR validation revealed an overexpression of the *FZD6* gene (a putative WNT5A receptor) in distinct clusters of LECs isolated from diabetic compared to otherwise healthy human eyes [73]. 

Taken together, alterations in the limbal morphology, LESC biomarker expression and their strong associations with corneal epithelial defects in diabetes emphasize that the limbal compartment and its resident cells play an important role in the pathogenesis of DK. However, since many of the current, well-accepted markers for LESCs also detect cells in other regions of the ocular surface, such as the conjunctiva, and/or cannot accurately discriminate bona fide SCs from early TACs [74], alternative investigations into their dynamics, density, and distribution are warranted to pinpoint their location in order to define the molecular and biochemical pathways that can be targeted for remedial action in disease.

## 4. Corneal Nerves, Immune Cells, and Their Interplay with LESCs in Diabetes

Nerve axons that infiltrate the cornea have multiple functions, one of which is to influence LESC activity. The causal relationship between corneal innervation and LESC function is well established in healthy mice [75]. In db/db mice, decreased intraepithelial corneal basal nerve (ICBN) parameters, including density, thickness and branchpoints (Figure 2), coincide with reduced HES1 and K19 mRNA and protein expression [33]. Abnormal corneal nerve parameters and diminished LESC marker expression also correlate with pathological features of DK, including irregular epithelial stratification and swelling, which is denoted by the presence of vacuoles within the corneal epithelia of diabetic mice [33]. Moreover, a loss of basal cell density and absence of the POV is associated with a significant reduction in the ICBN density in corneas of patients with T2D [62]. These findings suggest that LESC density and function are modulated by inherent alterations in corneal nerves due to hyperglycemia (Figure 1). However, the effects imposed by LESCs on corneal nerves, and the specific stage at which this occurs, remain ill-defined. 

Recently, a link between the sympathetic nervous system and LESC function was reported in corneas from the STZ-induced T1D mouse model [67]. In this paradigm, corneal epithelial abnormalities were associated with intrinsic β2-adrenoreceptor (Adrb2)-mediated sympathetic over-activation and the growth of sympathetic nerve fibers, which is consequential to hyperglycemia (Figure 1). Importantly, the abnormal hyperstimulation of these nerves correlated with diminished LESC marker expression and was associated with a significant reduction in the number of proliferating limbal cells, following chemical exposure in T1D compared to normal mice [67]. These observations suggest that upon inflicting a corneal injury, defective LESC proliferation accounts for the delayed corneal wound-healing response in diabetes. Because ocular sympathetic nerves are predominantly distributed adjacent to SCs within the limbus, which contain Adrb2 receptors [67,76], modulation of their activity during metabolic imbalances will likely influence their steady state (Figure 2). 

Sensory nerves also interact with resident immune cells such as dendritic cells and T cells which patrol the cornea as first-line responders to microbial insurance and trauma by initiating effective responses to acute and chronic stimuli [77]. Therefore, clinical manifestations of DK such as abnormal innervation and reduced corneal sensitivity may additionally result from changes in the immune system, which in turn impact LESCs [46,78] (Figure 2). In a mouse model of prediabetes, generated by obesogenic or high-fat diet, a reduction in corneal nerve function and upregulation of inflammatory mediators precedes the onset of hyperglycemia; these perturbations are accompanied by a significant decline in neutrophil numbers within the limbus of high-fat-diet-fed mice compared to normal diet-fed control mice [78]. In contrast, upon mechanical abrasion to the corneal epithelium, significant neutrophil accumulation occurs in the limbus compared to the central cornea, suggesting that some leukocytes are unable to effectively migrate to the site of injury, and that limbal inflammation may be responsible for prolonged wound healing in DK [78]. High numbers of other immune cells, including dendritic cells, have also been observed (via IVCM) in patients with T2D, which correlated with the absence of LESC-harboring POV; however, their distribution within the cornea was not specified [62] (Figure 2). The regulation of LESCs by T and B lymphocytes was reported in non-obese diabetic and a severe combined immune deficiency disease (NOD/SCID) mouse model [46]. Interestingly, NOD/SCID mice exhibited a barely detectable expression of the putative LESC markers, GPHA2 and CD63, and concurrently displayed DK-associated features such as corneal epithelial thickening when compared to healthy controls [46]. However, such observations should be interpreted with caution, because it is unknown whether these features develop as a consequence of absent T and B-cells in SCID mice, due to the diabetic state, or both. Understanding the profound interplay between corneal nerves, corneolimbal immune cells and LESCs, and their functional significance in diabetic corneal disease is warranted but requires further elucidation.

The importance of corneal innervation in modifying LESC function in diabetic corneas is further highlighted by the concurrent restoration of nerve parameters, normalization of LESC marker expression and considerable improvements in epithelial regeneration, following treatment with various interventions (Figure 1, green box). In db/db mice, the retrobulbar administration of growth factors such as insulin-like growth factor-1 (IGF-1) significantly improved ICBN features, which was concomitant with increased staining for HES1 and K19 [33]. Similar results were reported upon the suppression of sympathetic nerve hyperactivity via the subconjunctival injection of Adrb2 receptor-specific antagonists and after reducing excess nerve fiber growth through the surgical ablation of sympathetic nerves in eyes of STZ-induced T1D mice [67]. These strategies restored K15, ∆Np63α and GPHA2 protein and mRNA expression (Figure 1), suggesting they contribute to LESC dysfunction. Moreover, treating TKE2 cells (a murine corneolimbal cell line with stem-like features) with the neuroprotective cytokine, ciliary neurotrophic factor (CNTF), as well as in vivo application of the same mitogen, increased the expression of several putative LESC markers including ABCG2, BMI1, TCF4 and ∆Np63α [79]. This treatment also regenerated corneal nerves and significantly improved mechanically induced corneal epithelial wounds in T1D mice [79], thus reinforcing the effect of corneal innervation on LESCs. Follow-up molecular investigations in the same model unveiled a link between reduced LESC marker expression and the defective regulation of norepinephrine-sonic hedgehog (Shh) [67] and Akt-STAT3 transcriptional pathways [79] (Figure 2), although the precise sequence of events resulting in this association was not fully delineated.

## 5. Differential MicroRNA Expression in the Diabetic Limbus

MicroRNAs (miRNAs) are short non-coding RNA molecules consisting of 18–25 nucleotides that bind complementary mRNAs to negatively regulate the expression of protein-coding genes after transcription [80]. Accumulating evidence from knockout and transgenic animals suggest that individual miRNAs and the molecular pathways they act upon are involved in the spatiotemporal regulation of diverse biological processes, including cell cycling, organogenesis, embryogenesis, tissue repair, energy metabolism and intercellular communication [81,82]. Aberrant miRNA expression is linked to numerous conditions, including cardiovascular, metabolic, and infectious diseases as well as cancer [83,84,85,86]. Recently, miRNAs have become an active area of research especially in the realm of therapeutics due to their ability to modulate the downstream effects of multiple genes, in contrast to traditional approaches which typically target one gene [87]. 

Both human and animal studies have identified miRNAs within the cornea which regulate a variety of homeostatic processes including cell differentiation [88], proliferation [89], migration [90], apoptosis [91] and SC maintenance [88]. They can also modulate pathophysiological processes including corneal wound healing [90,92,93,94,95], neovascularization [96,97], inflammation [98] and immune defense [99,100]. Reports of differential miRNA expression across anatomically distinct ocular surface regions, such as corneal, limbal and conjunctival compartments [101,102,103], suggests that targeting specific miRNAs may serve as amenable therapeutic options for correcting ocular defects. The role of various miRNAs in the regulation of LESCs have also been highlighted, such as the involvement of miRs-103/107 in the regulation of LESC-associated macropinocytosis and autophagy via the modulation of Rac1, PKC and cyclin-dependent kinase 5 signaling pathways [104]. Most recently, miRNAs residing within the limbal stromal extracellular vesicles have been shown to support the stemness and phenotype of these cells through the upregulation of putative SC marker-associated RNAs and increasing cell proliferation by targeting the Notch1 signaling pathway [105]. 

In the diabetic cornea, variations in the expression of several wound healing-related miRNAs have been reported. qPCR-validated microarray analysis revealed that the expression of 29 miRNAs, previously linked to cell migration, inflammation, and proliferation, were indeed altered in diabetic compared to non-diabetic human cadaveric corneas [106]. These observations imply that the impairment of these endogenous cellular processes account for corneal epithelial defects that develop in this disease. Of the 29 miRNAs, miR-146a, 21 and 424 were expressed at the highest levels, while miR-509-3p and 143 were most significantly downregulated in diabetes. Similarly, differential signatures for 20 other miRNAs were discovered using genome-wide sequencing between the limbus of control versus T1D and T2D patients [107]. Therefore, quantitative changes in limbal miRNA expression could be responsible for depleted LESC reserves in diabetes and serve as biomarkers of DK. Functional aspects of the two most differentially expressed miRNAs, and the evidence demonstrating their influence on LESCs in diabetes, are discussed below. 

### 5.1. miR-146a

miR-146a is a key modulator of the innate immune response and is associated with the pathogenesis of numerous autoimmune diseases [108]. Its higher expression in the diabetic limbus relative to the central cornea, as well as higher levels in diabetic vs. non-diabetic limbus [109], suggests that its overexpression contributes to LESC peculiarities (Figure 2). miR-146a upregulation also correlates with delayed cell migration, wound closure, a reduced expression of epithelial wound-healing mediators (p38 and EGFR) as well as downregulation of the putative LESC markers, Frizzled-7 and K15 in primary human LECs and organ-cultured whole corneas procured from patients with diabetes [109]. Given that the central cornea comprises terminally differentiated cells, these observations indicate that miR-146a overexpression negatively influences the migration and differentiation of LESCs, thereby contributing to corneal anomalies that develop in DK. miR-146a is also associated with an altered inflammatory mediator expression in LECs derived from diabetic human corneas [110]. Upstream regulators of the NF-κB pathway, including interleukin (IL)-1 receptor-associated kinase 1 (IRAK1) and tumor necrosis factor receptor (TNFR)-associated factor 6 (TRAF6), in conjunction with NF-κB-related chemokines (CXCL1, CXCL2, CXCL3) and cytokines (IL-1α, IL-1β, IL-6 and IL-8), are significantly repressed at the mRNA and protein levels as a consequence of miR-146a overexpression in diabetic LECs [110]. Thus, the inability of diabetic LECs to upregulate key cytokines and chemokines after wounding could be a mechanism which suppresses corneal epithelial regeneration during a state of metabolic imbalance. Taken together, it can be inferred that abnormal miR-146a overexpression serves to dampen limbal inflammation in the diabetic cornea, although this occurs at the expense of efficient wound healing. Hence, targeting miR-146a downregulation in the limbus could serve as a potential therapy for DK. 

### 5.2. miR-10b

miR-10b has been linked to SC migration, epithelial–mesenchymal transition, and cancer metastasis [84,111,112]. Although its physiological functions remain unclear, it is one of the most abundant miRNAs expressed in the normal limbal epithelium [101]. Moreover, a comparatively higher expression of miR-10b in basal limbal epithelia suggests its crucial role in supporting LESCs. Genome-wide sequence analyses on human corneas indicate that miR-10b is also one of the most differentially and highly expressed miRNAs in the diabetic limbus [107] (Figure 2). Relative to its basal specification in the healthy limbus, miR-10b overexpression in all layers of the diabetic limbal epithelium implies that LESC function is impacted by this spatial assertion. Furthermore, the complete separation of limbal miRNAs derived from T1D and T2D patients (with miR-10b being the most differentially expressed) made possible via principal component analysis and hierarchical clustering suggests that LESC function is hindered by different forms of diabetes [107]. It is likely that LESCs are affected by T1D more so than T2D, since the number of differentially expressed miRNAs was markedly increased in the former. This difference in miRNA profile may be attributed to the greater proportion of genetic polymorphisms that contribute to the pathogenesis of T1D, which can be used to discriminate this form of diabetes from T2D [113]. Consequently, repressing miR-10b within the limbus may also serve as a potential target for the treatment of DK.

## 6. Contribution of Exosomes

Exosomes are a subset of extracellular vesicles comprising cellular constituents released upon fusion with the plasma membrane. They mediate numerous biological functions, including homeostasis, signal transduction and intercellular communication [114]. Although a dearth of data exist concerning their function in the diabetic cornea, the differential expression of 28 proteins, as well as morphological abnormalities, have been detected with transmission electron microscopy (TEM) and the proteomic profiling of plasma-derived exosomes from patients with T2D who have been diagnosed with DK [115]. The differential expression of miRNAs in exosomes derived from diabetic compared to normal human limbal stromal keratocytes has also been reported [116]. Moreover, when compared to normal counterparts in the same study, the application of diabetic limbal-derived exosomes could not rescue cell proliferation nor normalize wound healing mediator (p-38, Akt and EGFR) and LESC marker (K15 and Frizzled-7) expression in in vitro wounded LECs and in ex vivo organ-cultured whole human corneas, suggesting that if their contents are incomplete and/or compromised, disturbances in LESC homeostasis will ensue and potentially persevere (Figure 2). 

Recent studies demonstrated the benefits of administering healthy SC-derived exosomes in improving the corneal healing response in diabetes. The subconjunctival injection of healthy bone marrow mesenchymal SC-derived exosomes in diabetic mice, inflicted with a central corneal epithelial injury, displayed a significant reduction in corneal wound-healing rate [117]. Treatment with exosomes extracted from mouse adipose-derived mesenchymal SCs, in the form of eyedrops in STZ-induced T1D mice, also promoted significant improvements in corneal wound healing, nerve regeneration and sensitivity [118]. Follow-up experiments confirmed that such changes are linked to a dendritic cell-mediated reduction in inflammation concomitant with activation of the nerve growth factor (NGF)/Tropomyosin receptor kinase A (TrkA) signaling pathway [118]. Notably, NGF and its receptor, TrkA, are specifically expressed in a rare population of cells within the basal limbal epithelia, and both are therefore regarded putative markers of LESCs [119]. The therapeutic benefits of SC-derived exosomes in conjunction with defects associated with exosomes from the diabetic limbus suggests that impairment of their contents negatively influences corneal dynamics. However, the direct effect of exosome treatment on LESC marker expression or activity was not explored in these studies. Therefore, further investigations concerning the relationship between metabolic imbalance and SC-derived exosomes are warranted to ascertain their mechanism of action in diabetic LESCs, especially pertaining to DK. 

## 7. SC Therapy in DK

SC therapies for treating diabetic corneal complications have also been considered, revealing promising results. The administration of hematopoietic and mesenchymal SCs promotes corneal epithelial regeneration in animal models of diabetes [38,39]. Moreover, transplanting hematopoietic SCs in a rat model of DK increases corneal epithelialization with fewer vacuolated cells relative to untreated diabetic counterparts [39]. Similarly, corneal wound healing and epithelial proliferation are heightened, and inflammation is attenuated upon transplantation of bone marrow-derived mesenchymal SCs on the ocular surface of T1D mice [38], thereby collectively highlighting the therapeutic potential of SCs in this disease. Mechanistically, these ameliorating effects are attributed to elevated tumor necrosis factor-α-stimulated gene/protein-6 (TSG-6) expression within the corneal epithelium, which is known to regulate ECM stability and cell migration [38]. Since ECM proteins serve as a scaffold to facilitate cell migration during wound healing, activation of this pathway could account for enhanced epithelial regeneration upon SC administration in DK. However, the influence of such non-ocular tissue-derived SC treatment on LESC content and function was not elaborated. 

## 8. Insights into the Distinguishing Features of LESCs in DK and LSCD

**Figure 4:**
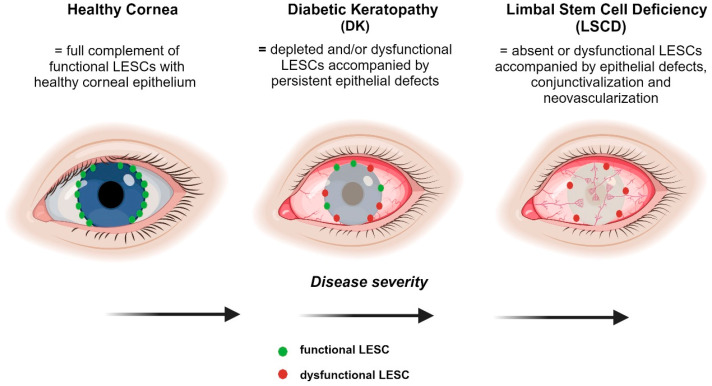
Comparison of the severity of LESC-associated defects in DK and LSCD. A healthy cornea comprises the full complement of functional LESCs with no epithelial defects. DK results in the depletion and/or dysfunction of LESCs with potentially some normal LESCs remaining. LSCD is characterized by the absence of functional LESCs which triggers conjunctivalization and neovascularization, resulting in opacification of the cornea. Figure generated with BioRender.com.

Despite current evidence highlighting the loss and/or dysfunction of LESCs in diabetes, there is scant in vivo functional data implicating these insufficiencies as being responsible for DK [62]. If it were the case, and because diabetes is a chronic systemic disease, then continuous loss of LESCs and disturbances in their activity are anticipated, potentially resulting in more severe manifestations similar to the pathological fibrovascular conjunctival tissue that enshrouds the cornea in LSCD [61]. Nonetheless, given that patients with early-stage diabetes do not meet diagnostic criteria for LSCD, regardless of LESC depletion [62], longitudinal studies are needed to identify diagnostic markers of this disease and the potential risks of developing LSCD over time. Conjunctivalization and neovascularization are two cardinal features of LSCD which have not been reported in patients with diabetes, even though they have been suggested [62]. However, severe non-resolving corneal epithelial defects are apparent in LSCD, which are similar to lesions that develop on the ocular surface of patients with diabetes [10,11,12]. Therefore, in diabetes, it is likely that a defined number, or indeed a specific population of LESCs, is depleted with sufficient stocks retained to preserve limbal barrier function and prevent conjunctival incursion into the cornea (Figure 4). Because epithelial defects on the corneal surface can persevere in DK, it is also tempting to speculate that LESC activity is exhausted, especially if their longevity is cut short by intrinsic factors that would otherwise be exquisitely regulated under homeostatic conditions. Moreover, unlike LSCD, LESC function in DK may be secondarily exacerbated as a consequence of compromised corneal sensitivity and innervation due to co-existing retinal conditions in patients with diabetes [120]. Notably, persistent epithelial defects such as neurotrophic keratopathy can develop after shaving or ablating the epithelium prior to retinal laser photocoagulation [121] or potentially due to thermal burns following laser refractive surgery [122] after which they may not fully regenerate [123]. Therefore, investigating such anomalies on a hyperglycemic background may shed new light into the pathobiology of LESCs within the diabetic cornea. Alternatively, metabolic inequities could modify the limbal microenvironment and encourage remaining LESCs to enter an abnormal state for which there are emerging biological, pharmacological, genetic, and other interventions to correct their status and restore ocular surface health and sight. 

## 9. Conclusions and Future Directions

It is evident that LESCs are influenced by a myriad of complexities that prevail systemically and locally in the diabetic cornea, ultimately resulting in their depletion or loss of function. Although the precise programs and molecular circuits that lead to the development of corneal defects in diabetes have not been fully elucidated, evidence suggests that defects in cell movement and the proliferation of LESCs exist. These processes are likely influenced by modifications to components of the limbal niche which have been imposed by a chronic hyperglycemic state. Direct links are established between LESC marker expression and corneal wound healing, innervation as well as inflammation in patients with diabetes and in representative animal models of this disease. Overall, these studies reveal the contribution of discoordinated healing-related signaling pathways, differentially expressed miRNAs and impairments in exosomes that impinge on LESC form and function (Figure 2). However, knowledge gaps exist concerning the precise sequence of molecular pathophysiological events leading to DK, and this continues to be a challenge, owing to the limited number of clinical studies that have been conducted in the field thus far. Future preclinical studies would be important for the identification and development of novel pharmacological targets for DK and their translation to the clinic. 

Specific gene-modifying strategies have further elucidated the importance of rescuing LESC function in treating diabetes-associated corneal defects. However, the clinical application of these techniques warrants further development to ensure side effects are minimized in the quest to restore LESC activity. Knowledge as to whether diabetes imposes major changes in the characteristic features displayed by LESCs, TACs or both populations and unravelling the detailed molecular pathways related to LESC dysfunction would be an invaluable undertaking to determine the efficacy of a therapeutic. Moreover, the pathological characteristics that distinguish the LESC phenotype in diabetes and LESC-associated diseases such as LSCD have not been explored. This would be imperative for developing advanced clinical management strategies and examining whether current diagnostic tools for LSCD can be exploited for diabetic corneal disease. With further confirmation of impaired LESC activity and knowledge on how treatment restores their density, a higher-order understanding of their role in diabetes would be gleaned. As biomarkers specific for DK are currently lacking, investigating clinical tools for the detection of changes in the limbal architecture and LESCs would be a key priority for improving the diagnosis of DK at early stages and advancing the future clinical management of ocular surface complications that arise in patients with diabetes. 

## Data Availability

The data presented in this study are available from the corresponding author upon reasonable request. Datasets are not publicly available due to privacy.

## Abbreviations

ABCG2	ATP-binding cassette superfamily G member 2
Adrb2	β2-adrenoreceptor
Akt	protein kinase B
BMI1	B lymphoma Mo-MLV insertion region 1 homolog
CD63	CD63 molecule
CF	cathepsin F
CNTF	ciliary neurotrophic factor
CXCL	C-X-C motif ligand
Db/db	diabetes/diabetes
DCN	diabetic corneal neuropathy
DK	diabetic keratopathy
DR	diabetic retinopathy
ECM	extracellular matrix
EGF	epidermal growth factor
EGFR	EGF receptor
GPHA2	glycoprotein hormone alpha-2
HES1	hairy and enhancer of split 1
ICBN	intraepithelial corneal basal nerve
IL	interleukin
IRAK1	Interleukin-1 receptor-associated kinase 1
IVCM	in vivo confocal microscopy
K	keratin
LDL	low-density lipoprotein
LEC	limbal epithelial cell
LESC	limbal epithelial stem cell
LSCD	limbal stem cell deficiency
miRNA	microRNA
MMP-10	matrix metalloproteinase-10
mRNA	messenger RNA
N-Cadherin	neural cadherin
NF-κB	nuclear factor kappa B
NGF	nerve growth factor
NOD	non-obese diabetic
Notch1	neurogenic locus notch homolog protein 1
PAX6	paired box protein 6
POV	palisades of Vogt
qPCR	quantitative polymerase chain reaction
Rac1	Ras-related C3 botulinum toxin substrate 1
RNA	ribonucleic acid
SC	stem cell
SCID	severe combined immunodeficient disease
scRNA-seq	single-cell RNA sequencing
Shh	sonic hedgehog
shRNA	small hairpin RNA
STAT3	signal transducer and activator of transcription 3
STZ	streptozotocin
T1D	type 1 diabetes
T2D	type 2 diabetes
TAC	transient amplifying cells
TCF4	transcription factor 4
TEM	transmission electron microscopy
TKE2	mouse corneal epithelial progenitor/stem-like cell line
TRAF6	tumor necrosis factor receptor associated factor 6
TrkA	tropomyosin receptor kinase A
TSG-6	tumor necrosis factor-α-stimulated gene/protein-6
Wnt	wingless-related integrated site
WNT5A	Wnt family member 5A
WT	wild-type
